# Persistence of Microbial Contamination on Transvaginal Ultrasound Probes despite Low-Level Disinfection Procedure

**DOI:** 10.1371/journal.pone.0093368

**Published:** 2014-04-02

**Authors:** Fatima M'Zali, Carole Bounizra, Sandrine Leroy, Yahia Mekki, Claudine Quentin-Noury, Michael Kann

**Affiliations:** 1 Université Bordeaux Segalen, Microbiologie Fondamentale et Pathogénicité Unité Mixte de Recherche 5234, Bordeaux, France; 2 Centre Hospitalier Universitaire de Nîmes, Service de Biostatistique, Epidémiologie Clinique, Santé Publique, Informatique Médicale, Nîmes, France; 3 Laboratoire de Virologie, Centre de Biologie et Pathologie Est, Hospices Civils de Lyon, Lyon, France; IPO, Inst Port Oncology, Portugal

## Abstract

**Aim of the Study:**

In many countries, Low Level Disinfection (LLD) of covered transvaginal ultrasound probes is recommended between patients' examinations. The aim of this study was to evaluate the antimicrobial efficacy of LLD under routine conditions on a range of microorganisms.

**Materials and Methods:**

Samples were taken over a six month period in a private French Radiology Center. 300 specimens derived from endovaginal ultrasound probes were analyzed after disinfection of the probe with wipes impregnated with a quaternary ammonium compound and chlorhexidine. Human papillomavirus (HPV) was sought in the first set of s100 samples, *Chlamydia trachomatis* and mycoplasmas were searched in the second set of 100 samples, bacteria and fungi in the third 100 set samples. HPV, *C. trachomatis* and mycoplasmas were detected by PCR amplification. PCR positive samples were subjected to a nuclease treatment before an additional PCR assay to assess the likely viable microorganisms. Bacteria and fungi were investigated by conventional methods.

**Results:**

A substantial persistence of microorganisms was observed on the disinfected probes: HPV DNA was found on 13% of the samples and 7% in nuclease-resistant form. *C. trachomatis* DNA was detected on 20% of the probes by primary PCR but only 2% after nuclease treatment, while mycoplasma DNA was amplified in 8% and 4%, respectively. Commensal and/or environmental bacterial flora was present on 86% of the probes, occasionally in mixed culture, and at various levels (10->3000 CFU/probe); *Staphylococcus aureus* was cultured from 4% of the probes (10-560 CFU/probe). No fungi were isolated.

**Conclusion:**

Our findings raise concerns about the efficacy of impregnated towels as a sole mean for disinfection of ultrasound probes. Although the ultrasound probes are used with disposable covers, our results highlight the potential risk of cross contamination between patients during ultrasound examination and emphasize the need for reviewing the disinfection procedure.

## Introduction

Endovaginal ultrasonography is commonly used in gynecology and obstetrics for investigation of suspected disease and pregnancy complications and for medically assisted procreation. Transvaginal as other endocavitary probes are considered semi-critical devices since they are not intended to penetrate skin or mucous membranes but only to come into contact with them. Being at lower risk of infection, sterilization of these equipments is neither required nor feasible. To minimize even further the risk, the endocavitary ultrasound probes are covered with a single use sheath, after coating the probe with a gel enabling sound transmission. Nevertheless, probe covers can fail, and probes can be contaminated by pathogens present in human secretions resulting in their nosocomial transmission. Thus, disinfection of the probes between patients is needed. However, there are no consensual guidelines for transvaginal probe disinfection. Health authorities such as the Centers for Disease Control and Prevention [1] or the American Institute of Ultrasound in Medicine [2] recommend a High-Level Disinfection (HLD) even for covered probes. HLD technologies consist of immersion in glutaraldehyde, hydrogen peroxide, or peracetic acid, and then rinsing and drying. They present many drawbacks such as possible deterioration of the transducer, chemical damage to the mucosa of patients and practitioners, toxic effects on the gametes and embryos, impaired imaging, and in all cases the time devoted to the procedure. As a consequence, current practice compliance with this standard is poorly followed [3], [4]. For this reason, other countries recommend a Low-Level Disinfection (LLD) procedure based on probe wiping with a single use towel (pre)impregnated with products such as quaternary ammonium compounds or phenolics [5]. Although very few cases or outbreaks of hospital acquired infections linked to endovaginal ultrasound procedures have been documented [6]–[9], the risk of cross infection must not be dismissed. Indeed, some reports have evidenced bacterial and/or viral contamination of LLD disinfected endovaginal probes [10]–[14]. Nevertheless, none, to our knowledge, has investigated contamination of vaginal ultrasound probes by both viruses and bacteria, including *Chlamydia trachomatis* and mycoplasmas, together with fungi.

While new HLD technologies for ultrasound probes such as gas plasma or ultraviolet C light systems are in evaluation to comply with current workflow [12], [15], [16], the question on whether there is a need to perform HLD between patients remains. The aim of this study was to assess the antimicrobial efficacy of the LLD procedure for transvaginal ultrasound probes on a range of potentially pathogenic microorganisms under routine conditions.

## Materials and Methods

### Study settings

Over a 6-month period (between April and September 2012) a prospective study was conducted in a large private French Radiology center. No patient information of any kind has been gathered, no human samples were tested in this study and the observed procedure complied with the national recommendations. Therefore no patient consent was required by the local ethical committee. A total of 300 consecutive samples were taken from vaginal ultrasound probes just after LLD disinfection of the probe.

### Standard disinfection procedure

The disinfection of the probes was performed by the clinician. Probes were covered with a medical CE mark disposable sheath. After examination, the probe cover was carefully removed to avoid probe contamination, and a visual inspection was performed to detect any break of the probe cover and any blood or body fluids on the probe. Then, the probe was cleaned with a non sterile dry tissue paper to eliminate the gel, and disinfected using wipes (Prodene, France) that are preimpregnated with a solution of ethanol/water, propylene glycol, myristalkonium chloride, menthol, and chlorhexidine digluconate.

### Sampling

Samples were taken from three endovaginal ultrasound probes using three ultrasound machines (Voluson E8, GE healthcare, USA) each one placed in a separate scanning room. All specimens were collected by a trained Microbiologist (F.M.), less than five minutes after the disinfection of the probes by the clinicians. The entire surface of the ultrasound probe was thoroughly sampled using flocked swabs (Copan Diagnostic, France). Swabs were immediately placed in transport media and brought to the laboratory. Delay between sampling and laboratory processing never exceeded three hours. The first 100 samples were analyzed for HPV detection, the next set of 100 samples for *C. trachomatis* and mycoplasmas, and the last set of 100 samples for bacterial and fungal screening. Specific flocked swabs and Universal Transport Medium (UTM-RT, Copan Diagnostic, France) were used for HPV, *C. trachomatis* and mycoplasmas, and eSwabs in Amies liquid (Copan Diagnostic, France) were used for the other microorganisms. For quality control purpose, samples were collected once a week from the ultrasound rooms using Count-Tact Agar plates (BioMérieux, France), and from the ultrasound gel bottles.

### DNA extraction and amplification

DNA was extracted from the samples using the semi-automated magnetic system NucliSENS, easyMag (BioMérieux, France) according to the manufacturer's instructions. PCR amplifications were carried out to screen for the presence of HPV, *C. trachomatis* and mycoplasmas using previously described primers and conditions [17]–[19]. All samples giving a PCR positive product were further subjected to a nuclease treatment to remove any free DNA and potentially leave likely viable microorganisms before another PCR assay was performed. The nuclease treatment consisted of mixing 900 μl of the sample with 2 μg Nuclease S7 (Sigma, France) in presence of 10 mM CaCl_2_ during 2 h at 37°C. The enzyme's activity was stopped by the addition of 30 mM EDTA. Each series included a negative control in order to test for contamination during the extraction procedure, and a positive control. One swab from each batch, as well as the transport medium batch, was tested for the absence of microbial contamination.

### Culture and identification of bacteria and fungi

Aliquots of 100 μl of the transport medium were spread on a series of agar plates (BioMérieux, France), either selective (Gardenella specific agar, Sabouraud agar) or not (Mueller Hinton agar, chocolate-polyvitex and horse blood agar). Plates were then incubated at 37°C overnight (Mueller Hinton agar plates), at 30°C for 2–5 days (Sabouraud plates), or at 37°C in a 5% CO_2_ enriched atmosphere for up to 48 h (chocolate-polyvitex, blood and Gardenella agar plates). After incubation, colony forming units (CFU) were enumerated. The ultrasound gel analysis was performed as above. Microorganisms grown on these plates and on the Count-Tact Agar plates were identified to species level and their antibiotic susceptibility was determined when relevant by conventional methods.

### Analysis

Statistical analysis of the number of pathogens was performed using the Stata 12/SE software (Statacorp LP, Texas). Data were expressed as number and 95% confidence interval (CI).

## Results and Discussion

The results of this study revealed that despite LLD, the ultrasound probes remained substantially contaminated by clinically significant microorganisms, including HPV, *C. trachomatis*, mycoplasmas, Gram-positive and Gram-negative bacteria. These results are in accordance with those of the few studies on this topic [10]–[14].

In the first subset of 100 samples screened by PCR for the presence of HPV, 13% (95% CI: 6–20) were positive. Such contamination rate is higher than previously described. For instance, Ma *et al.* [13] found that 7.5% of surveillance samples taken daily from vaginal transducers when the instrument was not in use, were positive for HPV DNA; interestingly, three of the 14 probe samples collected from HPV colonized patients were contaminated by HPV DNA. Casalegno *et al.* [11] reported 3.5% of HPV contaminated endovaginal probes, including 3% for at least one high risk (HR) type; furthermore, they detected HPV in 2.7% in pre-examination samples, including 1.9% of HR-HPV, and apparently the same HR-HPV persisted on an endovaginal probe despite three disinfection procedures. In both studies, endovaginal probes were used with similar probe covers and LLD procedure (wipes impregnated with quaternary ammonium compounds) as ours. HPV DNA was detected either by PCR covering more than 40 types of mucosal HPV [13] or by a microarray kit detecting 35 HPV genotypes [11], while the methodology used in the present study focused on 22 mucosal HPV genotypes. Thus, the lower rates of HPV contamination on disinfected endovaginal probes found in the literature might be due to differences in examined population, hygiene practice or, as suggested by some authors, the use of dry swabs resulting in a loss of sensitivity [11]. In an earlier study, Amis *et al.* [10] indicated that none of the condoms covered probes used for transvaginal sonography, wiped with a dry tissue and then with a 70% isopropyl alcohol towel were positive for herpesvirus but only few probes were examined (n = 26) and alcohol is known to shorten the working life of the probe. Recently, Kac *et al* [12] reported 1.5% of viral (Epstein-Barr virus, human cytomegalovirus and HPV) contamination on endovaginal/transrectal transducers after removal of the probe covers. After HLD using both disinfection with disinfectant impregnated towel and a 5-min cycle in an ultraviolet C chamber, no viral genome was detected.

In the second subset of 100 samples screened by PCR for the presence of *C. trachomatis* and mycoplasmas, 20% (95% CI: 12–28) and 8% (95% CI: 3–13) were positive, respectively. To our knowledge, no previous study has investigated the presence of these organisms on ultrasound transducers. Primers used for *C. trachomatis* detection amplify the cryptic 7.5-kb plasmid present in all serotypes; false negative reactions thus should only be encountered with the exceptional strains harboring a partly deleted plasmid or no plasmid at all [18]. PCR amplification used for *Mycoplasma* detection target 16S rRNA sequences that are genus-specific and react with all members of the genera *Mycoplasma* (including *Mycoplasma genitalium* and *Mycoplasma hominis*), *Ureaplasma* (in particular *Ureaplasma urealyticum*), *Spiroplasma* and *Acholeplasma* [19].

HPV cannot be propagated in tissue culture, and *C. trachomatis* and mycoplasmas are difficult to cultivate. Therefore, the accurate detection of these microorganisms in patients' samples relies on molecular biology techniques such as PCR amplifications, which are the most sensitive and specific tests [19]–[21]. However, DNA detection does not necessarily indicate the presence of viable and infective microorganisms. In an effort to select for infectious viral particles or bacteria, positive samples have been subjected to a second PCR amplification after DNase treatment. The percentages of positive samples fell by twofold for HPV (7%; 95% CI: 2–12) and mycoplasmas (4%; 95% CI: 0–8). A tenfold decrease was observed for *C. trachomatis* (2%, 95% CIX-X), may be reflecting both its high prevalence in female genital tract [20] and its limited survival in the environment and/or low resistance to disinfectants.

HPV is the most common sexually transmitted virus and is now recognized as the major etiological cause of invasive cervical cancer. *C. trachomatis* is the first agent of sexually transmissible diseases, and causes in women, cervicitis, pelvic inflammatory disease and its sequelae, i.e. infertility, ectopic pregnancy, and chronic pelvic pain [20]. “Genital mycoplasmas” are frequently isolated from the genital tract. *M. genitalium* is increasingly identified as the causative agent of pelvic inflammatory disease [22]; *M. hominis, and U. urealyticum* may induce a variety of genitourinary infections [23]. Perinatal transmission from mother to child has been demonstrated for HPV and *C. trachomatis*, the latter being responsible for neonatal conjunctivitis and pneumonia [20]. Genital mycoplasmas are involved in a number of adverse outcomes of pregnancy [23]. Considering the clinical impact of these pathogens their absence would have been desirable.

In the third subset of 100 samples taken for screening for bacterial/fungal contamination, no agent specifically responsible for sexually transmitted diseases such as *Neisseria gonorrhoeae,* or for vaginosis/vaginitis such as *Gardnerella vaginalis* and *Candida albicans* were found. In addition, organisms potentially deleterious for neonates, such as group B streptococci or *Escherichia coli*, were absent. Amis *et al*. [10] and Sykes *et al*. [14] did not evaluate the presence of *C. albicans*. Kac *et al.* [12] searched fungi and did not encounter any on endovaginal/transrectal probes even just after removal of the cover probe. None of them looked for gonococcus or gardnerella. Although all pathogenic organisms that can be transmitted through endovaginal ultrasonography [24] have not been investigated, the study has encompassed the most representative ones.

In contrast, 86% (95% CI: 79–93) of our samples, were contaminated by commensal and/or environmental bacterial flora, occasionally in mixed culture (Table 1). Skin flora, including coagulase-negative staphylococci (CNS, 73%), *Micrococcus* sp. (20%), methicillin susceptible *Staphylococcus aureus* (4%), viridans streptococci (2%), and *Corynebacterium* sp. (1%) was found predominantly and often in high numbers (10->3000 CFU/probe). Environmental flora (*Pseudomonas stutzeri, Shewanella putrefaciens* and *Aeromonas* sp. 2%; *Pseudomonas aeruginosa, Acinetobacter baumannii, Flavobacterium oryzihabitans*, and *Comamonas acidovorans*, 1%) was less represented and in lower amounts (10-90 CFU/probe). It is difficult to say which ones are the “pathogenic bacteria”. Indeed, as suggested by Koibuchi *et al*., [25] even coagulase-negative staphylococci and *Corynebacterium* spp. as some *Bacillus* spp. can cause critical infectious diseases in immunosuppressed patients. *S. aureus*, a part of the skin microbiota is one of the main causes of hospital-and community-acquired infections which can have serious consequences, and methicillino-resistant strains pose therapeutic issues [25]. Enterobacteria, which are the dominant aerobic flora of the digestive tract, may also be encountered [12]. Thus, outbreaks caused by *S. aureu*s and SHV-5 producing *Klebsiella pneumoniae* after endovaginal ultrasonography have been reported [6], [9]. Environmental flora, mainly composed of non-fermentative Gram-negative bacilli is responsible for nosocomial infections in debilitated patients. Outbreaks due to *P. aeruginosa* [26], [27], *Burkholderia cepacia* [28], [29], *Achromobacter xylosoxidans* [30] and recently multidrug resistant bacteria [31] have been increasingly associated with transrectal ultrasonography. These data indicate that full consideration of bacterial contamination of endocavity ultrasound probes is essential. Sykes *et al.,* [14] under similar conditions as in this study, observed that 83.3% of the samples from the transvaginal ultrasound equipment grew skin/environmental organisms, and 6.7% grew “potential pathogens”, one of which being *S. aureus*. Amis *et al.* [10] found only one of 46 transvaginal probes positive for bacteria (*Acinetobacter* spp.) using isopropyl alcohol wipes. Kac *et al*. [12] reported 3.4% of contamination by pathogenic bacteria on endovaginal/transrectal transducers, all of which disappeared after HLD.

**Table 1:** Quantification and identification of the bacterial flora present on the transducers.

Bacterial species	Number of positive samples	Estimated number of CFU on the probe per sample
**Gram positive cocci and bacilli**		
Coagulase-Negative Staphylococci	73	10->3000
*Micrococcus* sp.	20	10->3000
*Staphylococcus aureus*	4	10-560
*Streptococcus* sp.	2	10
*Corynebacterium* sp.	1	20
**Gram negative bacilli**		
*Pseudomonas stutzeri*	2	10–20
*Shewanella putrefaciens*	2	20–90
*Aeromonas* sp	2	30–40
*Flavobacterium oryzihabitans*	1	10
*Pseudomonas aeruginosa*	1	30
*Acinetobacter baumannii*	1	10
*Comamonas acidovorans*	1	20

The mechanism by which probe contamination occurs is unclear. With regard to specifically genital pathogens (HPV, *C. trachomatis*, mycoplasmas), an incidental perforation of the probe cover before/during the examination, or leakage of blood or secretions at the open rim of the sheaths might be involved. In our study, neither the damage of the cover nor the presence of blood or other body fluids on the probe after cover removal was detected by visual inspection. However, the possibility of contamination due to microscopic damage of the sheaths cannot be excluded. The Centers for Disease Control and Prevention and the American Institute of Ultrasound in Medicine recommend the use of condoms rather than cover probes because they are less prone to perforations (1–9% and up to 81% in one study) [1], [10]. However CE marked probe covers are preferred on the basis that condoms are not adapted to all types of transducers and may have a lower coverage of the heads. In the study of Kac *et al*. [12] both types of covers performed similarly. In this study, considering the high frequency and level of skin bacteria, manual contamination of the probes should not be excluded, although necessary precautions (e.g. trained personnel, use of gloves…) were taken. Alternatively, in this large urban Radiology center with a high frequency ultrasound usage, the probe may have been either inconsistently cleaned and disinfected, or sporadically contaminated after LLD procedure by the environment or by gloves previously in contact with the external genitalia [11], [13]. Samples taken from the ultrasound room and the ultrasound gel did not show significant microbial contamination.

In conclusion, this study demonstrates that a high proportion of endovaginal ultrasound probes remain contaminated despite the use of medically adapted probe covers and conventional LLD procedure. Therefore, these equipments actually could represent a potential vehicle for cross-transmission. To our knowledge there are no data on how many of these pathogens have to be inoculated in order to cause infection. Nevertheless, in order to prevent risks of cross contamination; it is advisable that the endovaginal ultrasound disinfection procedure is reviewed. More studies using other brands of probe covers, and disinfecting towels or novel decontaminating approaches are warranted.
